# Addressing Prompt Dependency in the Treatment of Challenging Behavior Maintained by Access to Tangible Items

**DOI:** 10.3390/bs14090828

**Published:** 2024-09-17

**Authors:** Jennifer R. Weyman, Madison Imler, Danielle A. Kelly

**Affiliations:** 1 Department of Special Education and Counseling, College of Education, California State University, Los Angeles, CA 90032, USA; 2 Department of Special Education, College of Education, University of Missouri, Columbia, MO 65211, USA

**Keywords:** challenging behavior, functional communication training, prompt dependency, tangible function

## Abstract

Prompt dependency is a common concern for individuals with developmental disabilities, particularly autism spectrum disorder. Previous research has shown that different interventions can be used to decrease prompt dependency. The purpose of the present study was to evaluate the efficacy of various treatments to decrease prompt dependency during functional communication training in the treatment of challenging behavior maintained by access to tangible items in a 16-year-old female diagnosed with autism spectrum disorder. Specifically, we compared the effects of differential reinforcement, vocal prompt fading, extended response intervals, and full physical prompts with a constant prompt delay to increase independent functional communication responses. The results of the study suggest that the prompt dependency treatment evaluation was efficacious in increasing independent functional communication responses and subsequently reducing challenging behavior to zero rates.

## 1. Introduction

Some individuals diagnosed with autism spectrum disorder engage in challenging behaviors. These challenging behaviors can manifest in a variety of ways, including self-injurious behavior (e.g., hitting or biting oneself, headbanging) and aggression (e.g., hitting, scratching, or biting others). More specifically, the prevalence of self-injurious behavior and aggression among individuals with autism spectrum disorder is 27.7% and 25%, respectively [1,2].

Challenging behavior can negatively impact the lives of individuals with autism spectrum disorder and their families. More specifically, individuals who engage in challenging behavior are more likely to receive behavior management interventions such as physical and mechanical restraint and are also more likely to be over-prescribed medication [3]. In addition, there are financial burdens associated with having a child who engages in challenging behavior [3]. Given the negative impact of challenging behavior in children with autism spectrum disorder, it is important to assess and subsequently develop an intervention to reduce the behavior. The best practice for assessing challenging behavior is to conduct a functional analysis [4]. The functional analysis is used to identify the variables that maintain challenging behavior [5]. It is an assessment tool used to identify the function or reason a behavior persists. The most common functions of challenging behavior identified via a functional analysis include escape from demands, automatic reinforcement, and access to attention or tangible items [6].

After identifying the function of challenging behavior, a function-based treatment tailored to the specific function can be developed to reduce the behavior. Functional communication training [7] is one of the most common treatments for challenging behavior [8]. Functional communication training involves providing the maintaining reinforcer following a functional communication response (FCR; e.g., handing over a picture card, vocally requesting) and placing the challenging behavior on extinction (i.e., not providing the maintaining reinforcer following challenging behavior). 

It is important that the individual is proficient in engaging in the FCR to reduce challenging behavior. Ringdahl et al. [9] evaluated the effects of low-proficient and high-proficient FCRs on the rate of challenging behavior maintained by access to tangible items. The low-proficient FCRs were those that required more restrictive prompting such as modeling and physical prompts, and high-proficient FCRs were those that required only vocal prompts. The experimenters found that functional communication training with the high-proficient FCR was more effective than the low-proficient FCR. Falcomata et al. [10] extended those findings by evaluating the effects of low- and high-proficient FCRs across several functions of challenging behavior (i.e., escape, attention, and tangibles). The experimenters found mixed results across functions. More specifically, sometimes the high-proficient FCR resulted in lower levels of challenging behavior relative to the low-proficient FCR, and sometimes they had the same effect. Together, these studies suggest that clinicians should use high-proficient FCRs relative to low-proficient FCRs. However, the authors caution that other variables, such as the individual’s preference for the FCR, their history with the FCR, and the response effort required to engage in the FCR, may have influenced the outcomes. Nevertheless, it is important to ensure that individuals are proficient in engaging in their FCR to maintain low levels of challenging behavior. 

Various prompt and prompt fading strategies have been used to teach FCRs [8]. Prompts cue the learner to engage in correct responding during instruction, and prompt fading allows for the transfer of stimulus control from the prompt to the discriminative stimulus [11,12]. 

Unfortunately, prompt dependency is a common concern for individuals with developmental disabilities, particularly autism spectrum disorder [13]. Prompt dependency is present when correct responding consistently occurs after a prompt is provided [14,15]. That is, the prompt may exert stimulus control over the behavior rather than the discriminative stimulus. For example, instead of the light switch serving as a discriminative stimulus when the room is dark, the therapist delivering the vocal prompt, “Turn on the light”, may control the behavior of flipping the switch. 

Prompt dependency may decrease opportunities for independence because the learner relies on the presence of the controlling prompt to evoke responding [12,16]. Prompt dependency may be particularly problematic in the context of functional communication training because the therapist’s prompts may control FCRs rather than the discriminative stimulus. This may result in an increase in challenging behavior when the prompts are removed. 

Current evidence-based interventions for preventing prompt dependency include prompt fading, differential reinforcement, and extended response intervals [12]. Prompt fading involves systematically removing the prompt until correct responding is evoked by the natural discriminative stimuli [17]. When using differential reinforcement as a treatment for prompt dependency, independent responses may receive higher quality reinforcement than prompted responses [16]. Extended response intervals involve waiting an extended duration of time without prompting for the learner to initiate an independent response [12]. The purpose of these interventions is to increase the likelihood that the individual engages in an independent response rather than wait to respond after the prompt.

Although there is literature on strategies to prevent prompt dependency, there is limited research on strategies to increase independent responding after the learner has already displayed prompt dependency for that response [12]. Gorgan and Kodak [12] used an adapted alternating-treatments design to evaluate the effects of vocal prompt fading, differential reinforcement, and extended response intervals to reduce prompt dependency in three individuals diagnosed with autism spectrum disorder or a developmental disability. The skills they targeted were responses that the participants reliably completed following a prompt, but not independently (e.g., doing laundry, cleaning the bathroom, tacting). They found that extended response intervals were most effective and efficient for the first participant, differential reinforcement was most effective and efficient for the second participant, and both vocal prompt fading and extended response intervals were most effective and efficient for the third participant. This suggests that the treatments designed to prevent prompt dependency can also be used to treat existing prompt dependency, and that these interventions should be tailored to each individual.

Although there is literature on strategies to prevent and treat prompt dependency, to the authors’ knowledge, no research has specifically addressed prompt dependency in the context of functional communication training to reduce severe challenging behavior. Thus, the purpose of this study was to evaluate the effects of differential reinforcement, vocal prompt fading, extended response intervals, and full physical prompts with a constant prompt delay on independent FCRs to address prompt dependency in the treatment of challenging behavior maintained by access to tangible items.

## 2. Method

### 2.1. Participant, Setting, and Materials

Scarlett was a 16-year-old White female diagnosed with autism spectrum disorder requiring substantial support, an intellectual disability, and a language delay. She was in a self-contained classroom with a one-on-one aide throughout the day due to her intensive behavioral needs. She also received 6 hr of behavioral therapy per week to assess and treat her challenging behavior (i.e., self-injurious behavior). She engaged in limited to no verbal communication; however, she was being taught to use an augmented and alternative communication device (i.e., Proloquo2Go on an iPad) to communicate. She could follow only a few one-step instructions (e.g., “touch your nose”, “stack cups”). In addition, she needed assistance to engage in daily living tasks (e.g., wiping after bowel movements, putting on clothing). 

Sessions took place in a university-affiliated clinic in a therapy room with a one-way mirror. All sessions were conducted by trained students enrolled in a graduate program for applied behavior analysis, under the supervision of a Board Certified Behavior Analyst. The room contained a table, two chairs, and was equipped with a video recording system. The materials for the study included a helmet, preferred toys as well as an iPad equipped with the Proloquo2Go app that she would use to communicate.

Prior to the initiation of sessions, Scarlett’s parents provided written informed consent to participate in the study. In addition, the therapists conducted a consultation with Scarlett’s parents to identify the challenging behaviors of concern and identify preferred items to include in subsequent analyses. During the consultation, the therapists used the Open-Ended Functional Assessment Interview [18]. The consultation suggested the possibility of multiple functions including attention, escape from demands or social avoidance, and access to tangible items. Prior to the initiation of the current study, a traditional functional analysis similar to Iwata et al. [5] with attention, alone, play, and escape conditions was conducted. In addition, a tangible condition was included. Finally, a social avoidance pairwise functional analysis similar to Harper et al. [19] was also conducted. The results suggested that Scarlett engaged in challenging behavior to gain access to tangible items and to escape social interactions. In the current study, a tangible pairwise functional analysis was conducted to confirm whether there was a tangible function. 

The tangible pairwise functional analysis (baseline) was conducted in one day. Functional communication training was conducted across four days averaging 6.3 sessions per day (range, 4 to 10). The prompt dependency treatment evaluation was conducted across six days averaging 5.3 sessions per day (range, 1 to 9).

### 2.2. Response Measurement

Trained data collectors gathered data on video recordings using BDataPro [20]. For ease of data collection, data recorders were allowed to review the videos several times before collecting data. Data were collected on self-injurious behavior, independent functional communication responses (FCRs), and prompted FCRs. Self-injurious behavior was defined as forceful contact of an open or closed hand on any portion of the body hitting her groin, closure of the upper and lower teeth on the flesh of any portion of the body, fingernails making contact with skin and dragging on any portion of the body, Scarlett’s buttocks forcefully lifting from surface and slamming back down, or moving torso in a backwards/forwards motion with enough force to move the chair, and grabbing of the helmet and shoving it in a downwards motion to her neck or mouth. Hitting toward her groin was excluded from the operational definition for self-injurious behavior because it exclusively occurred when she was on or about to start her menstrual cycle and was hypothesized to be related to discomfort. Independent FCRs were defined as pressing the “Toys, please” icon on her augmented and alternative communication device (i.e., Proloquo2Go on an iPad) prior to a therapist prompt. Prompted FCRs were defined as pressing the “Toys, please” icon on the iPad after a prompt (i.e., vocal prompt, vocal prompt while pointing to the button, full physical prompt, or eye nudge prompt) was provided by the therapist.

### 2.3. Interobserver Agreement and Procedural Fidelity 

Interobserver agreement (IOA) was calculated for 66.3% of sessions with a minimum of 33% in each phase and condition. The IOA was calculated by dividing the session into 10-s intervals and dividing the smaller number of the recorded behavior by the larger number of the recorded behavior in each interval, averaging the results, and multiplying by 100. During the tangible pairwise functional analysis (baseline), the mean IOA was 91.4% (range, 79.17% to 98.33%) for challenging behavior and 100% for independent FCRs. During functional communication training, mean IOA was 99.83% (range, 96.67% to 100%) for challenging behavior, 99.16% (range, 93.33% to 100%) for independent FCRs, and 97.58% (range, 91.67% to 100%) for prompted FCRs. During the prompt dependency treatment evaluation, mean IOA was 98.89% (range, 93.33% to 100%) for challenging behavior, 96.94% (range, 86.67% to 100%) for independent FCRs, and 91.94% (range, 63.33% to 100%) for prompted FCRs. During the extended response interval phases, the mean IOA was 98.82% (range, 93.33% to 100%) for challenging behavior, 92.53% (range, 66.67% to 100%) for independent FCRs, and 99.82% (range, 96.67% to 100%) for prompted FCRs. During the return to baseline, the mean IOA was 91.94% (range, 81.39% to 100%) for challenging behavior and 100% for independent FCRs.

Procedural fidelity was calculated for 42.1% (range, 33.3% to 66.7%) of sessions across phases and conditions. During baseline and the reversal to baseline, data were collected on variables such as whether toys were removed at the onset of the session and whether the item was provided contingent on challenging behavior. During functional communication training, data were collected on variables such as whether challenging behavior was ignored (e.g., attention or tangible items were not delivered contingent on self-injurious behavior) and whether the item was provided contingent on an FCR. During the prompt dependency treatment evaluation, data were collected on variables such as whether the correct prompt was provided and if challenging behavior was ignored. During the extended response interval phases, data were collected on variables such as whether no prompt was provided, and challenging behavior was ignored. Procedural fidelity was calculated by dividing the total number of correct steps by the number of incorrect and correct steps and multiplying by 100. The average procedural fidelity score across all phases was 94.7% (range, 50% to 100%). 

### 2.4. Pre-Experimental Procedures

A multiple stimulus without replacement preference assessment (MSWO) [21] was conducted to identify high- and moderately preferred items to include during the tangible pairwise functional analysis. Eight items were placed in a line in front of Scarlett, and Scarlett was instructed to pick her favorite. After Scarlett made a selection, she gained access to the item for 30 s. After 30 s, the item initially selected was removed and not replaced, the remaining items were rearranged, and Scarlett was again requested to pick her favorite. This was conducted until all items had been chosen. The highest preferred item was animal toys, and a moderately preferred item was beads. These items were included in all subsequent analyses.

### 2.5. Procedures and Experimental Design

A multielement design was used to analyze experimental control during the pairwise tangible functional analysis and prompt dependency treatment evaluation, and a reversal design was used to evaluate the effects of functional communication on challenging behavior. Across all phases of the study, Scarlett wore a helmet to reduce the impact of head-directed self-injurious behavior, and the helmet was removed at session 97. Therapists did not block self-injurious behavior across sessions. All sessions were 5 min. 

### 2.6. Tangible Pairwise Functional Analysis (Baseline)

A tangible pairwise functional analysis was conducted to identify if challenging behavior was maintained in part by access to tangible items. That is, the therapists alternated between one control condition and two test conditions. During the control condition, Scarlett had access to one highly preferred item (i.e., animal toys) and one moderately preferred item (i.e., beads) based on the results of the MSWO. Two items were included in an attempt to avoid satiation. There were no programmed consequences for challenging behavior. During the test condition, Scarlett received 2-min pre-session access to the preferred tangible items, and at the start of the session, the therapist removed the items. Contingent on challenging behavior, the therapist provided access to the tangible items for 30 s. 

### 2.7. Functional Communication Training 

Functional communication training was conducted to decrease challenging behavior and increase independent communication. The FCR (i.e., pressing “Toys, please” on an iPad) was identified via a discussion with the first author and the participant’s mother. Prompted or independent FCRs resulted in access to the preferred items for 30 s and challenging behavior was placed on extinction (i.e., challenging behavior did not result in access to the tangible items). A progressive prompt delay was used to teach the FCR. Prompts were increased across sessions. Initially prompts were provided at a 0-s delay, then a 1-s delay, and subsequently increased by multiplying the delay by 1.5 and rounding to the nearest whole number similar to Weyman et al. [22].

Prior to each session, a rule was provided (i.e., “If you want your toys, you can press, toys please”). At the onset of the session, the therapist removed the preferred tangible items, and a second therapist provided the prompt (i.e., stating “Toys, please” while pointing to the button) at the appropriate delay. A vocal prompt was used because it was the least intrusive prompt that would evoke a correct response, and vocal prompts were used in her clinical programming outside of challenging behavior reduction sessions. After Scarlett engaged in the FCR, she was provided the preferred tangible items for 30 s. After the prompt was faded out, Scarlett stopped engaging in communication. At session 22, the therapists probed the effects of an eye nudge prompt provided at an 18-s delay. The eye nudge did not increase responding (i.e., zero responses per minute of independent or prompted FCRs); therefore, the progressive prompt delay was restarted at session 23 with a prompt delay of 18 s using the original prompt. The 18-s delay was implemented across three sessions to reestablish FCRs prior to implementing prompt fading. Across all functional communication training sessions, if the participant did not engage in any prompted or independent FCRs, the session was terminated after 5 min. 

### 2.8. Prompt Dependency Treatment Evaluation 

The prompt dependency treatment evaluation was conducted to increase independent FCRs and decrease prompted FCRs. Four conditions were compared in a multielement design: extended response interval, differential reinforcement, full physical prompt, and vocal prompt fading. Conditions were randomized across each series (i.e., one iteration of each of the four conditions). During each condition, therapists wore different-colored shirts to promote discriminated responding. Prior to each session, the rule was provided (i.e., “If you want your toys, you can press, toys please”). In addition, during these conditions, challenging behavior was placed on extinction. If the participant did not engage in any prompted or independent FCRs, the session was terminated after 5 min. 

#### 2.8.1. Extended Response Interval

During the extended response interval condition, the therapist removed the preferred tangible items and contingent on an independent FCR, Scarlett would receive the tangible items for 30 s. The therapist did not provide any prompts during this condition.

#### 2.8.2. Differential Reinforcement

During the differential reinforcement condition, the therapist removed the preferred tangible items and contingent on an independent FCR, she would receive the tangible items for 30 s as well as therapist attention throughout the 30-s period. If Scarlett did not engage in an independent FCR within 15 s of the tangible item removal, the therapist provided a vocal prompt (i.e., “Press the button”). A 15-s delay was used to allow for the opportunity for independent responding. Contingent on a prompted FCR, Scarlett received the tangible items for 30 s but did not receive therapist attention. 

#### 2.8.3. Full Physical Prompt

During the full physical prompt condition, the therapist removed the preferred tangible items and contingent on an independent FCR, Scarlett would receive the tangible items for 30 s. In addition, a constant prompt delay was used. If Scarlett did not engage in an independent FCR within 15 s of the toy removal, the therapist gently picked up Scarlett’s finger to guide her to press the button. A 15-s delay was used to allow for the opportunity for independent responding. Contingent on a prompted FCR, Scarlett received the tangible items for 30 s. It is important to note that physical prompts were not typically used in Scarlett’s clinical programming; therefore, we gained verbal consent to use physical prompts from her parents prior to implementing this condition. We evaluated this condition because based on a previous assessment, Scarlett engaged in challenging behavior to avoid social interactions. We hypothesized that she would engage in the FCR independently to avoid a full physical prompt. 

#### 2.8.4. Vocal Prompt Fading

During the vocal prompt fading condition, the therapist removed the preferred tangible items. Following an independent FCR, Scarlett would receive the tangible items for 30 s. If Scarlett did not engage in an independent FCR within 15 s of the tangible item removal, the therapist provided a vocal prompt (“Press the button”). A 15-s delay was used to provide an opportunity for independent responding. Contingent on a prompted FCR, Scarlett received the tangible items for 30 s. Across sessions, the vocal prompt was faded out. Fading was implemented as follows: “Press the button”, “Press the b”, “Press”, “P”, and then no prompt. Each prompt level was implemented once before transitioning to the next prompt. If Scarlett did not engage in independent FCRs after the prompt was fully faded out for one session, vocal prompt fading was restarted with the original prompt, “Press the button”, in the following session.

### 2.9. Extended Response Interval and Reversal

Following the prompt dependency treatment evaluation, an extended response interval phase was implemented to determine whether independent FCRs would maintain when conditions involving therapist prompts were removed. The procedures were identical to the extended response interval condition of the prompt dependency treatment evaluation. Following this phase, a reversal to baseline was conducted and the extended response interval phase was reintroduced. 

## 3. Results

**Figure 1:**
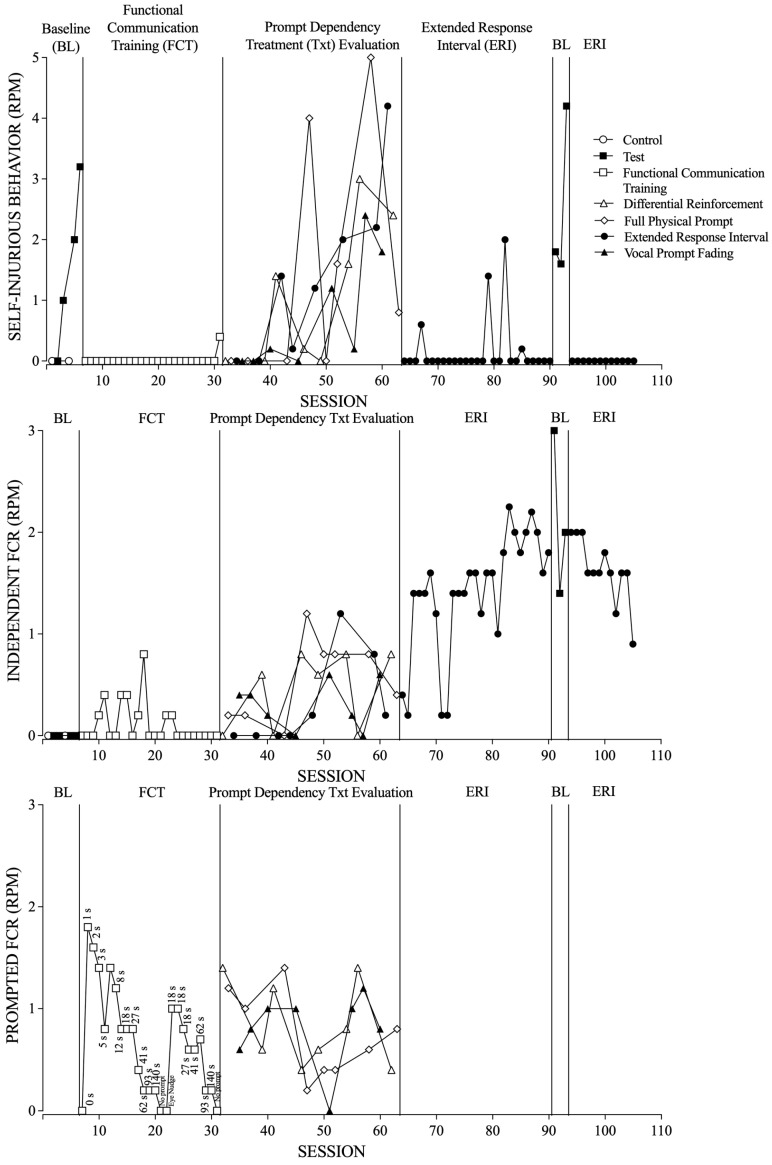
The rate of challenging behavior (**top** panel), independent FCRs (**middle** Panel), and prompted FCRs (**bottom** panel).

Figure 1 displays the rate of challenging behavior (top panel), independent FCRs (middle panel), and prompted FCRs (bottom panel) across all phases of the study. 

### 3.1. Tangible Pairwise Functional Analysis (Baseline)

During the tangible pairwise functional analysis (baseline), Scarlett engaged in a higher rate of challenging behavior during the test condition relative to the control condition, suggesting her challenging behavior was maintained in part by access to tangible items. Independent FCRs were not observed during any of these sessions. 

### 3.2. Functional Communication Training

During FCT, Scarlett engaged in zero rates of challenging behavior except in the final session in which she engaged 0.4 responses per min of challenging behavior. Scarlett engaged in a variable rate of independent FCRs, and she stopped engaging in independent FCRs during the final eight sessions. In regard to prompted FCRs, as the progressive prompt delay was increased, Scarlett engaged in fewer rates of prompted FCRs. At session 22, an eye nudge prompt at an 18-s prompt delay was probed, but did not result in an increase in independent FCRs. Next, the progressive prompt delay was restarted at session 23 with a prompt delay of 18 s and the original prompt. As the progressive prompt delay was increased again, Scarlett engaged in fewer rates of prompted FCRs and independent FCRs did not increase. These results suggest that Scarlett was prompt dependent (i.e., responding occurred following the prompt, but not independently). 

### 3.3. Prompt Dependency Treatment Evaluation

#### 3.3.1. Extended Response Interval

During the extended response interval condition, there was an increasing trend in challenging behavior. In regard to independent FCRs, Scarlett initially engaged in zero rates of independent FCRs; however, in the second half of the phase, starting at session 48, independent FCRs increased, and a decreasing trend occurred toward the end of the phase.

In addition, the average rates of challenging behavior, independent FCRs, and prompted FCRs were calculated across conditions. During the extended response interval condition, the average rate of challenging behavior was 1.4 responses per min (range, 0 to 4.2), and the average rate of independent FCRs was 0.3 responses per min (range, 0 to 1.2).

#### 3.3.2. Differential Reinforcement

During the differential reinforcement condition, there was an increasing trend in challenging behavior. Scarlett engaged in variable rates of independent FCRs, with higher rates observed during the second half of the treatment evaluation. There was a moderate and variable rate of prompted FCRs. 

The average rate of challenging behavior was 1.1 responses per min (range, 0 to 2.4), the average rate of independent FCRs was 0.5 responses per min (range, 0 to 0.8), and the average rate of prompted FCRs was 0.9 responses per min (range, 0.4 to 1.4).

#### 3.3.3. Full Physical Prompt

During the full physical prompt condition, there were variable rates of challenging behavior. In regard to independent FCRs, Scarlett initially engaged in low rates, but the rate increased during the second half of the treatment evaluation, starting at session 47. There was a moderate rate of prompted FCRs with a slight increasing trend toward the end of the treatment evaluation. 

The average rate of challenging behavior was 1.4 responses per min (range, 0 to 4), the average rate of independent FCRs was 0.6 responses per min (range, 0 to 1.2), and the average rate of prompted FCRs was 0.8 responses per min (range, 0.2 to 1.4).

#### 3.3.4. Vocal Prompt Fading

During the vocal prompt fading condition, there were moderate levels of challenging behavior with an increasing trend. Scarlett engaged in low to moderate rates of independent FCRs, and there was a moderate rate of prompted FCRs.

The average rate of challenging behavior was 0.7 responses per min (range, 0 to 2.4), the average rate of independent FCRs was 0.3 responses per min (range, 0 to 0.6), and the average rate of prompted FCRs was 0.8 responses per min (range, 0 to 1.2).

### 3.4. Extended Response Interval and Reversal

Following the prompt dependency treatment evaluation, therapists implemented an extended response interval phase. Scarlett engaged in low rates of challenging behavior, except during a few sessions. Scarlett also engaged in a high rate of independent FCRs. A reversal to baseline was implemented, resulting in a high rate of both challenging behavior and independent FCRs. Following the reintroduction of the extended response interval condition, challenging behavior decreased to zero rates and independent FCRs remained high.

## 4. Discussion

The results of the current study extend research on the treatment of prompt dependency in the context of reducing challenging behavior. More specifically, the purpose of the current study was to assess the effects of various treatments for prompt dependency on independent FCRs to gain access to tangible items in the treatment of challenging behavior maintained by access to tangible items. The current study suggests that the isolated and potentially combined effects of differential reinforcement and full physical prompts with a constant prompt delay were effective in increasing independent FCRs. In addition, after the independent FCR was acquired, challenging behavior was reduced. 

During the prompt dependency treatment evaluation, elevated rates of independent FCRs were observed during the differential reinforcement and full physical prompt conditions and moderate rates of independent FCRs were observed during the vocal prompt fading condition. In addition, during the extended response interval condition, rates of independent FCRs began at zero and increased to high/moderate but variable rates toward the end of the phase. It is possible that the independent effects of the differential reinforcement and the full physical prompt conditions resulted in high rates of independent FCRs. However, it is also possible that the combined effects of these conditions resulted in a high rate of independent FCRs, and these effects carried over to the extended response interval condition. Although the therapists attempted to combat multiple treatment interference and carryover effects by using discriminative stimuli during each of the prompt dependency conditions (i.e., therapists wore different-colored shirts), it is unknown if the same effects would be observed if the conditions were more isolated.

It is interesting to note that Scarlett engaged in a high rate of independent FCRs during the full physical prompt condition. It is possible that the use of a full physical prompt was effective because in addition to engaging in challenging behavior to gain access to tangible items, Scarlett also engaged in challenging behavior to avoid social interactions as determined by a previous functional behavior assessment. This may have increased the effectiveness of using full physical prompts. 

Functional communication training is one of the most common treatments for challenging behavior [8]. However, there is limited research on how to modify functional communication training when the individual also engages in prompt dependency. The current study demonstrates different methods to increase independent FCRs when the individual engages in prompt dependency. In addition, it demonstrates that functional communication training (i.e., reinforcing a FCR and placing challenging behavior on extinction) is a viable treatment for those who engage in challenging behavior maintained by access to tangible items and engage in prompt dependency.

Differential reinforcement has been an efficacious method of increasing independent responding for individuals with a history of engaging in prompt dependency [12,16]. The results of the current study replicate the findings of Cividini-Motta and Ahearn [16] as well as Gorgan and Kodak [12]. In the current study, independent responses resulted in 30-s access to the tangible item and therapist attention, whereas prompted responses resulted in only 30-s access to the tangible item. Future researchers could evaluate other parameters of reinforcement (e.g., duration of tangible item access, quantity of tangible items) in the context of differential reinforcement to decrease prompt dependency. 

Extended response intervals have been effective in treating prompt dependency [12]. Toward the end of prompt dependency treatment evaluation in the current study, Scarlett started to engage in a high/moderate rate of independent responding during the extended response interval condition. In addition, independent responding maintained during the extended response intervals phase following the prompt dependency treatment evaluation. Although it is possible that this was a result of carryover effects, it is also possible that the intervention itself was efficacious. These results add to the limited research on using extended response intervals to treat prompt dependency. Future research should continue to evaluate the effects of extended response intervals in the treatment of prompt dependency. Additionally, it is important to conceptualize the difference between extended response intervals as a treatment and functional communication training. In the current study, the extended response interval condition is identical to functional communication training in which an FCR is reinforced, and challenging behavior is placed on extinction. Although the procedures in the current study are identical, the rationale behind the procedures are slightly different. Generally, the goal of both procedures is to increase appropriate behavior and decrease challenging behavior. However, a secondary goal of the extended response intervals is to remove the contingency between the prompt and the FCRs. 

Prompt fading has also been shown to treat prompt dependency [12,23]. In the current study, vocal prompt fading was moderately effective. That is, it resulted in moderate to low levels of independent FCRs. These findings add to the existing literature on using vocal prompt fading to address prompt dependency. 

There are several limitations of the current study. First, the current study was only conducted with one participant which limits the generality of the findings. Second, there may have been multiple treatment interference within the multielement design during the prompt dependency treatment evaluation. A multielement design may not have been the best choice in experimental designs given that the behavior being taught (i.e., independent FCRs) was the same across conditions. A third limitation is that a helmet was placed on the participant’s head to reduce the impact of head-directed self-injurious behavior across the majority of sessions. It is unknown how the helmet influenced responding throughout this study. A fourth limitation is that although the prompt dependency treatment evaluation resulted in independent FCRs, it also resulted in a high rate of challenging behavior throughout the evaluation. Future researchers may evaluate these procedures using a latency-based measure to reduce the overall rate of challenging behavior. 

Although there are limitations, the prompt dependency treatment evaluation did result in a high rate of independent FCRs, and ultimately, challenging behavior was reduced to zero rates. This suggests that the procedures in the current study did reduce prompt dependency. Given there is limited research on strategies to address prompt dependency after it has been established, the current study provides some positive evidence that prompt dependency can be addressed in the treatment of challenging behavior. Future research may evaluate the effects of these treatments to target a variety of skills in which individuals exhibit prompt dependence. 

## Data Availability

The data that support the findings of this study are available from the corresponding author upon reasonable request.
